# The design and function of birds' nests

**DOI:** 10.1002/ece3.1054

**Published:** 2014-09-24

**Authors:** Mark C Mainwaring, Ian R Hartley, Marcel M Lambrechts, D Charles Deeming

**Affiliations:** 1 Department of Biological Sciences, Macquarie UniversitySydney, NSW, 2109, Australia; 2 Lancaster Environment Centre, Lancaster UniversityLancaster, LA1 4YQ, U.K; 3 Centre d'Ecologie Fonctionnelle et Evolutive, CEFE UMR 5175, Campus CNRS1919 Route de Mende, F-34293, Montpellier Cedex 5, France; 4 School of Life Sciences, University of LincolnRiseholme, Park, Lincoln, LN2 2LG, U.K

**Keywords:** Architecture, behavior, environmental adjustment, evolution, host–parasite coevolution, natural selection, nest, sexual selection

## Abstract

All birds construct nests in which to lay eggs and/or raise offspring. Traditionally, it was thought that natural selection and the requirement to minimize the risk of predation determined the design of completed nests. However, it is becoming increasingly apparent that sexual selection also influences nest design. This is an important development as while species such as bowerbirds build structures that are extended phenotypic signals whose sole purpose is to attract a mate, nests contain eggs and/or offspring, thereby suggesting a direct trade-off between the conflicting requirements of natural and sexual selection. Nest design also varies adaptively in order to both minimize the detrimental effects of parasites and to create a suitable microclimate for parents and developing offspring in relation to predictable variation in environmental conditions. Our understanding of the design and function of birds' nests has increased considerably in recent years, and the evidence suggests that nests have four nonmutually exclusive functions. Consequently, we conclude that the design of birds' nests is far more sophisticated than previously realized and that nests are multifunctional structures that have important fitness consequences for the builder/s.

## Introduction

Nest building is a taxonomically widespread activity, with birds, mammals, reptiles, fish, and insects all constructing nests of some description in which to lay eggs and/or raise offspring (Hansell 2000). There is a huge amount of variation in nest design across taxa, with nests varying from underground burrows dug by mammals, minimal nest scrapes on the ground in which game birds lay their eggs, the craters constructed by fish on the bed of water bodies, the huge mounds constructed by termites through to the cup-shaped nests of song birds in trees and bushes (Collias and Collias 1984; Reichman and Smith 1990; Hansell 2005). Even within taxa, there is a great deal of variation in nest design and in birds, nests range from the small but elaborate cup-shaped nests built by passerine birds through to the huge mounds built by megapodes (Hansell 2000).

Nest design varies considerably between and even within taxa, yet all nests have the same basic, minimal, function which is to provide a receptacle in which animals can lay their eggs and/or raise their developing offspring (Heenan 2013). This rather simplistic view of nests has generally prevailed over the years, and studies examining the function of birds' nests have been scarce, particularly when compared to other stages of reproduction (Lessells 1991; Hansell 2005). Illustratively, a study of six commonly studied nestbox-breeding passerine birds showed that fewer than 6% of 676 published studies reported any aspect of nest characteristics, despite researchers using nestbox-breeding birds as model systems due to the ease with which their reproductive parameters can be quantified (Lambrechts et al. 2010). This oversight is unfortunate as there is considerable evidence that nests are sophisticated structures that require considerable cognitive abilities to construct (Collias 1986; Muth and Healy 2011; Walsh et al. 2011). Fortunately, our understanding of the design and function of birds' nests has increased considerably in recent years, and here, we review the functions of birds' nests. We begin by examining the influence of natural and sexual selection, before examining the influence of parasites and environmental variation in determining nest-building behaviors and nest design.

## Natural Selection

Avoiding predation is a ubiquitous challenge for most birds, and natural selection favors those individuals with effective antipredator defenses (Caro 2005). Natural selection exerts selective pressures not only on the design of nests, but also on the birds themselves during the nest-building period while they are collecting and transporting material to the nest site (see review in Lima 2009). Accordingly, there are a number of ways in which the design of nests can minimize the risk of predation, including the location in which nests are built.

### Nest site selection

The selection of a safe nesting site is an important determinant of reproductive success, and some birds have been shown to choose their nest sites in order to reduce the risk of predation. An observational study showed that dusky warblers (*Phylloscopus fuscatus*) selected safer nest sites that were farther from the ground and in more isolated bushes when predatory Siberian chipmunks (*Tamias sibiricus*) were abundant, despite such locations carrying costs in terms of higher exposure to cold winds (Forstmeier and Weiss 2004). Elsewhere, veeries (*Catharus fuscescens*) selected nest sites with low levels of predatory white-footed mice (*Peromyscus leucopus*) activity (Schmidt et al. 2006), and Inca Terns (*Larosterna inca*) showed a clear preference for inaccessible crevices on cliffs that suffered lower predation rates than more exposed cliff sites (Verlando and Márquez 2002). These observational studies have also been supplemented with experimental studies. Experimentally placing wasp (*Polybia rejecta*) nests in close proximity to rufous-naped wren (*Campylorhynchus rufinucha*) nests resulted in experimental wren pairs suffering significantly lower rates of predation from white-faced monkeys (*Cebus capucinus*) than control pairs without wasps close by, as the monkeys actively avoided the wasps (Joyce 1993). When the calls of predatory corvids were played in Siberian jay (*Perisoreus infaustus*) nesting areas, the jays responded by nesting in safer, but less well insulted, sites (Eggers et al. 2006). Orange-crowned warblers (*Vermivora celata*) responded to novel nest predator playbacks by shifting from nesting in trees and shrubs nesting on to the ground (Peluc et al. 2008). In summary, it appears that local abundance of predators does result in adaptive shifts in nest site selection, with birds' nesting in safer locations when the abundance of predators is high. Such shifts in nest sites are presumably under strong selection pressures as such shifts often entail costs through reduced thermoregulatory benefits in sites with lower levels of predation risk.

The threat of predation has resulted in some animals nesting in association with more aggressive species, whose heightened antipredator defenses also benefit the focal species (see review in Quinn and Ueta 2008). Illustratively, breeding choughs (*Pyrrhocorax pyrrhocorax*) associate with lesser kestrels (*Falco naumanni*) and benefit through the kestrels being very vigilant for, and aggressive toward, potential nest predators. As the kestrels do not prey upon the choughs, then the association is entirely beneficial for the choughs as they suffer significantly fewer nest predation events and consequently have higher levels of breeding success when compared to conspecifics breeding without an association to the kestrels (Blanco and Tella 1997). However, not all associations may be so advantageous, and in other instances, the protective species can sometimes also prey upon the protected species (Caro 2005), which means that there may be an optimal nesting distance between them. Nest predation rates suffered by red-breasted geese (*Branta ruficollis*) are generally negatively correlated with their distance to more aggressive peregrine falcon (*Falco peregrinus*) nests. However, the geese are also harassed or attacked by the falcons if they nest too close, meaning that the geese optimally nest at least 40–50 m away from the falcons (Quinn and Kokorev 2002). Nesting associations are therefore an effective way of reducing the threat of predation upon nests (Quinn and Ueta 2008).

Density-dependent patterns of nest predation are also expected to affect the spacing of nests, and while there is a general consensus that nest predation rates increase as nest density increases, there are too few empirical studies to confirm this (Caro 2005). One notable exception comes from a study of mustelids, rodents, and colonially nesting fieldfares (*Turdus pilaris*). Mustelids favor rodent prey but shift to the contents of fieldfare nests when rodents are scarce. Consequently, mustelid predation on fieldfare nests increases as rodent density decreases, and there was a clear tendency for fieldfare colonies to form during years of low rodent abundance and for nesting to be more dispersed or noncolonial, during high rodent years. Hence, colonially nesting birds provided more effective mobbing defenses against mustelid predators, and it was suggested that the fieldfares track rodent density directly as a surrogate cue of predation risk (Hogstad 1995). While this study strongly suggests that predation can alter the optimal spacing of nests, this is clearly an area where further research is warranted.

The height of nests from the ground also influences nest predation rates (Martin 1993; Lima 2009). In a controlled experiment using artificial nests that represented the nests of open-cup nesting passerine birds, it was shown that higher nests were predated significantly more often than nests placed on the ground. The higher nests were predated by avian predators, meaning that the ground nests were safer despite them being more at risk from a range of mammalian predators (Piper and Catterall 2004). Elsewhere, lesser kestrels preferentially occupied holes located high up on churches as predation rates were negatively correlated with the height of the nest from the ground (Negro and Hiraldo 1993). Interestingly, the height of Oahu Elepaio (*Chasiempis ibidis*) nests on the island of Hawaii were negatively correlated with their risk of predation from introduced black rats (*Rattus rattus*) and the height of nests increased by more than 50% between 1996 and 2011, which led to a decline in nest predation rates (Vanderwerf 2012). By contrast, higher long-tailed tit (*Aegithalos caudatus*) nests were predated more frequently by avian predators, such as jays (*Garrulus glandarius*) and magpies (*Pica pica*), than lower nests (Hatchwell et al. 1999). Consequently, there is good evidence to suggest that birds vary the height at which they build their nests in response to predators as they build their nests higher from the ground in response to mammalian predators and lower in response to avian predators. Further, birds should also adapt following a predation event, and there is evidence that if a parent survives a nest predation event, then they disperse further distances to begin another nesting attempt when compared to a successful nesting attempt (review in Lima 2009). For example, female goldeneyes (*Bucephala clangula*) whose nests were predated by pine martens (*Martes martes*) were twice as likely to nest in new locations the following year, than females whose nests were not predated (Dow and Fredga 1983). To our knowledge, no studies have examined dispersal distances of focal individuals in relation to the breeding success of neighboring conspecifics, and further studies could usefully examine this issue.

### Nest design

The design of completed nests also influences the risk of predation (Caro 2005), and for example, ground-nesting birds must rely on crypsis to conceal their nests from predators. A study of Japanese quail (*Coturnix japonica*) found that egg patterning and color varied between, but not within, females and individual females consistently selected those laying substrates that matched the patterning and color of their eggs to make the visual detection of their eggs most challenging for predators. This suggests that the quail “knew” their individual egg patterning and color and actively sought out a nest site that provided the most effective camouflage (Lovell et al. 2013). Some birds cover their eggs in the absence of an incubating parent, and a study of mallard ducks (*Anas platyrhynchos*) found that when nests were covered with nest material, they suffered significantly lower rates of nest predation than nests which were left experimentally uncovered (Kreisinger and Albrecht 2008). However, while such behaviors may improve the crypsis functions of nests, there is often an assumption that crypsis is traded off against the requirement to create optimal microclimates within the nests (Lima 2009). This trade-off was examined in a study of little grebes (*Tachybaptus ruficollis*) which lay their eggs on floating nests built from wet plant material and cover their eggs with surplus nesting material when no parent is incubating. When nests were experimentally left uncovered, they suffered both lower predation rates and reduced temperatures when compared to control nests that were left covered (Prokop and Trnka 2011), thereby providing no evidence for such a trade-off. However, further research could usefully examine the potential trade-off between the requirements of crypsis and thermoregulation in animals.

The risk of predation also influences the design of nests that are built above ground. Darwin's small tree finch (*Camarhynchus parvuus*) females preferred to pair with males that built nests that were well concealed by surrounding vegetation, whereas exposed nests were rarely used for nesting (Kleindorfer 2007). Elsewhere, larger eastern olivaceous warbler (*Hippolais pallida elaeca*) nests were predated significantly more often than smaller nests (Antonov 2004). However, observational studies examining nest sizes and predation rates may be confounded by nest site selection, clutch sizes, and parental activities, and several studies have attempted to disentangle these potential determinants of nest predation. Nest predation rates are extremely high in the tropics, and one study examined whether higher nest predation rates select for smaller nests. When nests of different sizes were experimentally swapped around, nest predation rates increased with nest size, but not with the location of nests, indicating that nest size was the primary determinant of nest predation (Biancucci and Martin 2010). An experimental study showed that artificially enlarged blackbird (*Turdus merula*) nests were predated more frequently than nests which remained unchanged in size and nests which were made artificially smaller (Møller 1990a). Meanwhile, a study which examined the relative contributions of nest size, nest site, parental nest defense behaviors, and clutch size in determining the predation rates upon blackbird nests found that higher nests and nests with greater external diameters were predated more often than expected by chance (Gregoire et al. 2003). Elsewhere, nest failure in blackbirds was found to be dependent on the nest's detectability, and to a lesser extent, height, but not on parental behaviors, clutch size, or nest site characteristics (Cresswell 1997). Therefore, it appears that nest predation rates are not solely explained by either their size or location, and further studies are required in order to elucidate their relative contributions to nest predation.

In summary, the requirement to minimize the risk of predation strongly influences both nest site selection and the design of completed nests. However, while there is strong evidence that nest predation rates influence nest design over evolutionary timescales, few studies have examined whether nest design varies adaptively within a bird's lifetime (Lima 2009). This omission has presumably occurred because of the general assumption that nest building is a largely instinctive process (Hansell and Ruxton 2008; Raby and Clayton 2009). Consequently, further studies examining changes in nest building in responsive to variable levels of predation risk a bird's lifetime would be valuable.

## Sexual Selection

Nest design is strongly influenced by natural selection, yet nests may also be extended phenotypic signals of the builder/s quality and hence also be influenced by sexual selection. Individuals normally signal their quality through physical or behavioral signals such as brightly colored wing patches, elaborate songs, or extravagant ornaments such as crests (Andersson 1982), yet some species build external structures that signal their phenotypic quality (Schaedelin and Taborsky 2009). Species such as bowerbirds (Madden 2003) build structures whose sole purpose is to attract a mate (Schaedelin and Taborsky 2009). By contrast, nests usually contain eggs and/or offspring, thereby potentially suggesting a direct trade-off between the conflicting requirements of natural and sexual selection (Moreno 2012). However, for nest construction behaviors and nest design to be extended phenotypic signals that play a role in sexual selection, they must reliably indicate the quality of the builder by being associated with costs (Andersson 1982; Maynard Smith and Harper 2003).

### Nests as extended phenotypic signals

The process of fetching material to construct nests intuitively appears costly, yet such potential costs have generally been overlooked as they were often assumed to be negligible, particularly when compared to the costs of producing eggs or provisioning offspring (Dolnik 1991; Hansell 2005; Heenan 2013). However, there is a growing awareness that the costs of constructing a nest are far higher than previously imagined and indirect evidence of such costs comes from behaviors suggesting that animals minimize the costs of nest construction. Illustratively, some species exploit the efforts of others by stealing nesting material or completed nests from conspecifics (Moreno et al. 1995; Lindell 1996) or heterospecifics (Ewins et al. 1994; Schulz 1997), while other species breed in old nests despite incurring costs due to ectoparasitism (Brown and Brown 1986; Møller 1990b).

Direct evidence that nest building is a costly process comes from studies that have estimated the costs, with one study showing that cliff swallows (*Petrochelidon pyrrhonota*) constructing a 600 g nest expend 122 kJ by making an estimated 1400 trips to collect construction materials (Withers 1977). Meanwhile, observational evidence that nest construction behaviors accurately reflect the quality of the builder/s comes from studies which report a positive correlation between the phenotype of the building parent and the time to completion of nests (Lens et al. 1994; De Neve and Soler 2002) and the size of completed nests (Moreno et al. 1994; Lens et al. 1994; Fargallo et al. 2001; Tomás et al. 2006; Mainwaring et al. 2008). Elsewhere, a comparative analysis by Soler et al. (2007) found a positive correlation between nest-building effort and immunity both among European passerine birds and among barn swallows (*Hirundo rustica*), thereby demonstrating that birds in higher body condition invested more energy in nest building.

Experimental studies have also demonstrated that nest building is costly. For example, male Australian reed warblers (*Acrocephalus australis*) build multiple nests within their territories, which consist of one “type 1” nest that is structurally capable of holding eggs and nestlings and one or more “type 2” nests that are not structurally capable of holding eggs and nestlings. When experimental males were provided with supplementary food, they built more “type 2” nests within their territories than unfed control males (Berg et al. 2006). Meanwhile, supplementary fed female blue tits (*Cyanistes caeruleus*) built heavier nests than unfed control females in one study (Mainwaring and Hartley 2009) and shallower nests in another study (Smith et al. 2012), while great tit nest sizes did not differ between treatments (Smith et al. 2012). Therefore, despite none of these studies demonstrating any advantages of larger nests to the builders, nest-building behaviors appear to be limited by the availability of food. Other studies have further tested the costs of nest construction by directly manipulating nest-building effort, rather than carrying out indirect manipulations of food availability. When the nests and eggs of experimental pairs of pied flycatchers (*Ficedula hypoleuca*) were removed, thereby forcing them to build a second nest, experimental females built smaller nests than control females (Moreno et al. 2008). Further, when the costs of nest building were experimentally reduced in pied flycatchers, experimental females spent more time incubating their eggs before provisioning their nestlings at a higher rate than control females. This resulted in nestlings in experimental nests having longer tarsi at prefledging than nestlings in control nests (Moreno et al. 2010), although the advantages accrued by having longer tarsi are presently unclear (Mainwaring and Hartley 2012). Meanwhile, Lambrechts et al. (2012) experimentally removed the nests and eggs of blue tits after about 5 days of incubation and found that experimental females, which were forced to expend effort by building a second nest, built smaller nests and laid smaller clutches than control females.

To summarize, there is now observational, comparative, and experimental evidence that nest construction is a costly process. While these studies have focused disproportionately on birds, presumably because their nest-building visits can be accurately counted and their completed nests can be weighed and measured (Mainwaring and Hartley 2013), there is no reason to suggest that nest construction is not associated with costs in other taxa (Barber 2013). There is strong evidence that nest construction is costly (Maynard Smith and Harper 2003; Schaedelin and Taborsky 2009), and so nest-building behaviors and nest design have the capacity to act as extended phenotypic signals, which may be influenced by sexual selection (Andersson 1982; Moreno 2012).

### Male-built nests

Observational studies have shown that male-built nests play a role in sexual selection (Andersson 1982; Soler et al. 1998a,b) as there is often a positive correlation between some aspect of nest size or design and some aspect of the male's phenotype. In penduline tits (*Remiz pendulinus*), those males that constructed larger nests were more successful in acquiring a female (Hoi et al. 1994, 1996). Although male care was not correlated with nest size, females invested more care into broods raised in large nests, meaning that males that built large nests benefited through increased reproductive success (Szentirmai et al. 2005).

These observational studies have also been supplemented by several experimental studies. Male black wheatears (*Oenanthe leucura*) carry about 2 kg of stones to their nest sites, and observational studies have shown that males with larger wing areas carry more stones to nesting sites than males with smaller wing areas (Møller et al. 1995). The wing area of males appears to be a morphological adaptation to carrying such stones as when some of the primary feathers of experimental males were removed, they carried fewer stones to their nests than control males (Møller et al. 1995). Nest sites contain a mixture of old and new stones, and when old stones were experimentally removed from nests, males did not respond by carrying more stones, implying that females choose males on the number of new stones that they transfer to nesting sites before each breeding attempt (Soler et al. 1996). Further, when new stones were experimentally added to nest sites during the stone-carrying period, males carried fewer stones to nests, and when stones were experimentally removed, males compensated by carrying more stones to the nesting site (Moreno et al. 1994; Soler et al. 1996). In an experiment which manipulated the number of stones at nests, it was found that females which were paired with males that carried more stones responded by laying earlier in the breeding season, which led to experimental pairs having higher reproductive success than control pairs (Moreno et al. 1994; Møller et al. 1995; Soler et al. 1996).

In starlings (*Sturnus vulgaris*) and spotless starlings (*Sturnus unicolor*), males build the nest almost entirely alone, while females occasionally add feathers to nests. Both species incorporate green plant material into their nests, and the function of such material is thought to be associated either with sexual selection or with limiting the detrimental effects of ectoparasites. In spotless starlings, the majority of green plant material was carried to nests during the ten days prior to the start of egg laying, and those males that carried more green plant material to nests controlled a larger number of boxes, meaning that they had more female partners (Veiga et al. 2006). Meanwhile, the experimental removal and addition of green plant material had no effect on ectoparasite abundance or the mass of nestlings in starling nests, although males with experimentally increased amounts of green plant material did attract females more successfully than control males (Brouwer and Komdeur 2004). When the amount of green plant material was experimentally increased in spotless starling nests to mimic increased male nest-building effort, females responded by carrying more feathers to nests. Such responsive building behaviors were interpreted as functionally related signaling behaviors that played an important role in courtship activities and the signaling of status (Polo and Veiga 2006). Further, the experimental addition of green plant material in spotless starling nests resulted in females laying larger clutches (López-Rull and Gil 2009) and the skewing the sex ratio of such broods by increasing the number of sons in those eggs (Polo et al. 2004). Consequently, these studies show that the function of green plant material within starling and spotless starling nests is to play a role in sexual selection rather than as an antiparasite behavior.

Meanwhile, the females of some species choose males based on the number of nests that they build within their territories (Metz 1991). In birds, studies have shown that males that build more nests within their territories have greater reproductive success in yellow-shouldered widowbirds (*Euplectes macrourus*) (Savalli 1994), red bishops (*Euplectes orix*) (Friedl and Klump 2000) and wrens (*Troglodytes troglodytes*) (Garson 1980; Evans and Burn 1996; Evans 1997a,b). Despite one study reporting that the experimental addition of nests did not increase the pairing success of male marsh wrens (*Cistothorus palustris*) (Leonard and Picman 1987), there is good evidence that males that build multiple nests gain increased reproductive success. More generally, there is strong evidence that male-built nests act as signals to females, who adjust their reproductive investment accordingly (Andersson 1982; Moreno 2012).

### Female-built nests

Meanwhile, there is a growing appreciation that female-built nests reflect the phenotype of the building female in a similar way to male-built nests (Moreno et al. 2008, 2010; Lambrechts et al. 2012). However, studies examining the function of female-built nests are less common than studies of male-built nests for two reasons. First, female-built nests are considered to be relatively uncommon when compared to male-built and bi-parentally built nests (Collias and Collias 1984; Hansell 2000), and second, the theory of extended phenotypic signals has focused disproportionately on male signals (Andersson 1982; Moreno 2012).

Observational studies of female-built nests are relatively scarce, but one study showed that female spotless starlings, which placed feathers in their nests within nestboxes whenever they were locally available, did so in a nonrandom manor (Veiga and Polo 2005). Wood pigeon (*Columba palumbus*) and spotless starling feathers that show higher ultraviolet and visible reflectance on their reverse side were overwhelmingly placed with this side upwards, jay feathers which have higher reflectance on the obverse side were overwhelmingly placed with this side upwards, while azure-winged magpie (*Cyanopica cyana*) feathers were placed randomly as both sides have similar reflectance values. This indicates that feathers were placed so that their conspicuousness was maximized and suggests that they play a role in sexual selection (Veiga and Polo 2005). In blue tits meanwhile, healthier females that were less infected with *Trypanosoma avium* built heavier nests than females that had higher infection rates (Tomás et al. 2006). While it is generally acknowledged that female blue tits build the nest alone, a recent study examined why males sometimes carry feathers into nests. Males that delivered feathers had longer tarsi and fed their offspring more frequently than males that did not deliver feathers, and females responded to the delivery of feathers by reducing their own provisioning rates. Nevertheless, the females still obtained direct fitness benefits as the nestlings fledged in better condition than nestlings in those nests where males did not carry feathers to the nest (Sanz and García-Navas 2011). When feathers were experimentally added to blue tit nests, thereby mimicking nest building by extra-pair males, social males responded to such uncertainty over their own paternity by reducing the frequency at which they provisioned the offspring and in their nest defense behavior, when compared to control males (García-Navas et al. 2013). In ecologically similar great tits (*Parus major*) meanwhile, the phenotypic quality of females did not correlate with nest size or characteristics (Álvarez and Barba 2008), but nest size and characteristics were positively correlated with reproductive success (Alabrudzińska et al. 2003; Álvarez and Barba 2011). This discrepancy may be explained by another study which found that females with relatively high chromatic breast plumage, and not body size *per se*, built bigger nests and particularly so when paired to males with relatively high chromatic breast plumage (Broggi and Senar 2009). Elsewhere, young female tree swallows (*Tachycineta bicolor*) built nests with fewer feathers and had reduced fledging success when compared to older females (Lombardo 1994), suggesting that experience may play a part in determining variation in nest design.

Experimental studies of female-built nests are rare, but one study examined how the amount of green plant material placed in blue tit nests influenced male behavior. When the size of nests and the amount of green plant material were experimentally enlarged or reduced, male risk-taking behaviors were found to be significantly lower at those nests reduced in size and significantly higher at nests where green plants were added. Males that exhibited increased risk-taking behaviors at nests with more green plant material resulted those pairs having increased reproductive success, meaning that females that placed more green plant material in their nests accrued fitness benefits via increased male investment (Tomás et al. 2013). In summary, the evidence to date suggests that female-built nests are extended phenotypic signals, but experimental studies examining these issues are generally lacking, and this is clearly an area where further research is warranted (Tomás et al. 2013; Moreno 2012).

### Bi-parentally built nests

Observational studies examining the function of nest-building behaviors in bi-parentally built nests are relatively uncommon because bi-parentally built nests are relatively uncommon when compared to both male-built and female-built nests (Collias and Collias 1984). In crested tits (*Parus cristatus*), only males in good condition contributed to nest building, which shortened the interval between the start of nest building and the onset of laying by about 5 days. This resulted in nestlings fledging about 5 days earlier and as earlier fledged nestlings had enhanced survival prospects, then male nest-building efforts increased offspring fitness (Lens et al. 1994). Studies of barn swallows have shown that higher quality males with long tails contributed less to nest construction than lower quality males with shorter tails. Female nest-building effort remained constant across males with varying tail lengths, yet females paired with males with longer tails built nests with thinner walls and larger nest cups so that they could lay larger clutches inside them (Soler et al. 1998a,b). Interestingly, male tail lengths have increased over temporal timescales as anthropogenic climate change has increased ambient temperatures and has led to a general reduction in male nest-building effort and a subsequent decline in nest size (Møller 2006). Similarly, female rufous bush robins (*Cercotrichas galactotes*) responded to greater male effort during the nest-building stage by laying larger clutches (Palamino et al. 1998). However, extended phenotypic signals may not always be an honest indicator of the builder's quality, and signaling theory suggests that such exaggeration should be punished (Moreno 2012). Objects placed in black kite (*Milvus migrans*) nests were found to be an honest indicator of the pair's phenotypic quality by accurately predicting their fighting ability. Black kite pairs settle to breed in territories containing suitable nesting sites, but nonbreeding birds sometimes attempt to violently take over such breeding territories. Nests containing many objects were built by pairs with high fighting capabilities and lower quality birds did not dishonestly signal their phenotypic quality. Such cheating would have easily been possible, but the honesty of this signal was maintained by the threat of individuals being severely hurt in aggressive challenges from intruding birds (Sergio et al. 2011).

There are a few experimental studies which have examined how bi-parental nest-building behaviors are influenced by sexual selection. In chinstrap penguins (*Pygoscelis antarctica*), both sexes collect stones in order to protect their eggs and chicks against flooding, thereby suggesting that natural selection determines the collection of stones (Moreno et al. 1995). When nests were experimentally manipulated so that some had half of the stones removed, some had half of the stones removed and snow added, while control nests were left alone, the penguins at nests where stones were removed had increased stone-provisioning rates by 44%, and those nests with stone removal and snow added increased their stone provisioning by 123%, while control nests remained unaltered or unchanged. This indicates that stone carrying is determined by both sexual and natural selection (Fargallo et al. 2001). Female magpies (*Pica pica*) adjusted their reproductive effort in relation to the male's nest-building efforts. When the first clutches of experimental pairs were removed, high-quality pairs that originally built large nests were more capable of building a replacement nest, and females were found to lay larger clutches in nests that were built faster, irrespective of nest size (De Neve and Soler 2002). Moreover, a study which experimentally enlarged magpie nests, thereby mimicking increased male nest-building effort, resulted in females laying larger clutches and beginning incubation later, thereby creating fewer late hatched nestlings which have poor survival prospects (Soler et al. 2001). Interestingly, great spotted cuckoos (*Clamator glandarius*) preferentially parasitize those magpies which have built larger nests, as nest size provides a reliable indication of parental quality (Soler et al. 1995). As a consequence, magpies living in areas with great spotted cuckoos have been found to build smaller nests than magpies living in areas without cuckoos (Soler et al. 1999). Finally, a study examined the function of feather carrying behaviors in male house sparrows (*Passer domesticus*). Males call to females when adding feathers to the nest, suggesting that they wish the behavior to be noticed, and when feathers were experimentally removed from nests, males responded by carrying more feathers, although the number of feathers carried to nests varied between males. The volume of feathers delivered by males was positively correlated with clutch size and female provisioning rates. While this suggests that feathers play a role in sexual selection, the feathers were usually added during the incubation and the early nestling period, when the need for insulation was greatest. Consequently, feathers probably play a role in both sexual and natural selection in house sparrows (García-López de Hierro et al. 2013).

In summary, there is clear evidence to suggest that nests are extended phenotypic signals that accurately indicate the phenotypic quality of the building parent/s. This applies to male-built, female-built, and bi-parentally built nests (Moreno 2012) and is an important development as while species such as bowerbirds build structures that are extended phenotypic signals whose sole purpose is to attract a mate (Schaedelin and Taborsky 2009), nests contain eggs, and/or offspring, thereby suggesting a direct trade-off between the conflicting requirements of natural and sexual selection (Moreno 2012). A trade-off between natural and sexual selection is likely to occur because while natural selection selects for small nests, sexual selection selects for big nests. However, our current understanding of this trade-off is relatively poor, and further research is required to further understand how these conflicting requirements are resolved.

## Host–parasite coevolution

Parasites constitute more than half of all living species, and consequently, interactions between parasites and their hosts are among the most ubiquitous forms of interspecific interactions. Such interactions are usually characterized by a situation in which one organism, the parasite, lives on or in the body and benefits at the expense of the other organism, the host (Clayton and Moore 1997). Parasites often have considerable negative impacts on the fitness of their hosts, which leads to the hosts employing defenses against the parasites. This has resulted in a coevolutionary arms race where hosts and parasites have to change continuously simply to keep up with the other's adaptations (Loye and Zuk 1991; Clayton and Moore 1997).

### Parasites and host fitness

Birds host a variety of parasites including lice, fleas, mites, ticks, leeches, fungi, and bacteria, yet relatively little was known about the impact of such parasites on their hosts, aside from commercially valuable game birds, until the 1980s (Clayton and Moore 1997). Then, an influential paper by Hamilton and Zuk (1982) argued that the elaborate displays of a range of North American birds evolved as a consequence of parasite-mediated sexual selection, which led to an increased interest in the impacts of parasites on wild birds. Many studies have since shown that parasites can have severe consequences for their host's fitness by reducing their survival and reproductive success (Loye and Zuk 1991; Clayton and Moore 1997; Proctor 2003). For example, when the number of hen fleas (*Ceratophyllus gallinae*) was experimentally increased in great tit nests, it was found that when compared to control pairs, experimental pairs laid their eggs later in the season, the parents deserted their clutches more frequently during the incubation period and hatched and fledged fewer nestlings (Oppliger et al. 1994). Meanwhile, a study that experimentally increased the number of fowl mites (*Ornithonyssus bursa*) in multibrooded barn swallow nests found that when compared to control pairs, experimental pairs had lower reproductive success as indicated by a reduced number of independent fledglings from first clutches and reduced clutch sizes, brood sizes, and the number of independent fledglings from second clutches (Møller 1990b).

Given the negative effect of parasites, it is unsurprising that hosts have evolved a wide variety of defenses against them (Loye and Zuk 1991; Clayton and Moore 1997). In birds, such defenses include plumage maintenance behaviors such as molting feathers, the use of feather toxins, body maintenance behaviors such as preening and dusting, and a range of nest maintenance behaviors (Toft 1991; Loye and Zuk 1991).

### Nest design as a host defense

Many birds place green plant material and feathers in their nests and usually replenish them on a daily basis throughout the incubation and nestling stages of reproduction (Wimberger 1984; Brouwer and Komdeur 2004; Peralta-Sanchez et al. 2010). Green plant materials contain volatile secondary compounds such as hydrocarbons, mainly monoterpenes and isoprene, which could have biocidal effects on parasites and pathogens (Clark 1991; Brouwer and Komdeur 2004; Dubiec et al. 2013). Meanwhile, the majority of bacteria found on feathers are known to produce antibiotic substances, meaning that feathers could prevent the establishment of other bacteria within the nest environment (Peralta-Sanchez et al. 2010). Consequently, both green plant material and feathers may inhibit parasites, although they may also provide thermoregulatory benefits and/or play a role in sexual selection. For example, the nonbuilding partner may select mates on the quantity of feathers or green plant material placed in nests (Brouwer and Komdeur 2004; Peralta-Sanchez et al. 2010), and builders may use green plant material to signal their quality to their nonbuilding partners as in contrast to visual cues, fresh plant material in dark nests may be an olfactory cue of the builder's quality (Clark 1991). Consequently, there are several nonmutually exclusive hypotheses that seek to explain the function of feathers and green plant material in birds' nests, and they have been examined in a few species.

The function of green plant material has been well studied in starlings, where males build the nest alone. Males select only a small subset of available plant species and have been shown to prefer those plants that possess higher concentrations of mono- and sesquiterpenes than randomly available plant species (Clark and Mason 1985). While this suggests an antiparasite function, a study that removed green plant material from experimental nests found that when compared to control nests, experimental nests actually contained fewer ectoparasites and nestlings were heavier, although postfledging survival did not differ (Fauth et al. 1991). When all original nests were replaced with either experimental nests containing green plant material or control nests containing grass, it was found that ectoparasite abundance was similar between treatments (Gwinner et al. 2000). However, nestlings in nests containing green plant material were heavier and had higher hematocrit levels, and although fledging success was similar, postfledging survival was higher among nestlings raised in nests containing green plant material (Gwinner et al. 2000). These studies suggest, but do not provide conclusive evidence, that green plant material inhibits parasites. Further, the amount of green plant material carried to nests by males was positively correlated with the time taken to attract a female during courtship (Gwinner 1997). This suggests that green plant material plays a role in sexual selection, which was supported in a study that found that males nesting in nestboxes experimentally contaminated with ectoparasites did not carry more plant material to nests than males in control nestboxes (Brouwer and Komdeur 2004). Further, unpaired males carried more greenery to nests when a caged female was positioned adjacent to nests than when a caged male or an empty cage was present, while paired males did not respond to these cues (Brouwer and Komdeur 2004). Together, these studies suggest that male starlings place green plant material in their nests primarily to attract females and secondarily to repel ectoparasites.

In blue tits, where the females build the nest alone, the experimental addition of green plant material resulted in higher nestling masses in experimentally enlarged, but not reduced, broods (Mennerat et al. 2009a,b). Also aromatic plants significantly reduced bacterial richness on nestlings, but not on parents (Mennerat et al. 2009c). This suggests that green plant material serves to limit the effects of ectoparasites, which was partially supported in a study that added green plant material to experimental nests and grass to control nests. Fleas were less abundant and blackflies and midges more abundant in experimental nests built by young females, although nestling growth and immunity did not differ between treatments or with female age (Tomás et al. 2013). However, when the amount of aromatic plants within blue tit nests was experimentally increased, there was no decline in the number of Protocalliphora blow flies (Mennerat et al. 2008). When the nests of blue tits and pied flycatchers differing in composition were swapped between the two species, experimentally induced changes in nest composition did result in significant changes in the abundances of mites, fleas, and blowflies in both species (Moreno et al. 2009). Differences in ectoparasite abundances between the two bird species were maintained, whatever the experimental change in nest composition used. Meanwhile, blue tit nests have been shown to have a range of distinct odor classes that are easily perceived by humans (Lambrechts and dos Santos 2000), which occurs because green plant material contains chemical compounds used by humans to make aromatic house cleaners and herbal medicines (Petit et al. 2002). Blue tit parents use odor cues to determine when to replenish green plant material and both parents hesitated longer outside their own nestboxes when their nests had been experimentally supplied with fresh green plant material than when supplied with moss (Mennerat 2008). A further complication comes from a study that showed that ants occasionally occupy blue tit nests and their presence may modify host–parasite interactions (Lambrechts et al. 2008). It is presently unclear whether the ants exploit their avian hosts using their nests as places to search for ectoparasites, and there are still too few studies to completely discount the antiparasite functions of green plant material within blue tit nests, meaning that further studies are required.

The function of feathers within bird's nests has also been studied in tree swallows. When feathers were added to experimental nests, ectoparasites were more abundant in those nests than in control nests where feathers were left untouched, thereby providing no support for the hypothesis that feathers physically separate nestlings and ectoparasites (Dawson et al. 2011). In another study, nestlings in experimental nests where feathers were removed had higher infestations of mites and lice and lower growth rates when compared to nestlings in control nests. Consequently, there was no reduction in the nestling's exposure to ectoparasites, although the feathers did provide thermal benefits to the nestlings (Winkler 1993). Consequently, there is no evidence that feathers within tree swallow nests provide protection from ectoparasites, although one study added green plant material to experimental nests and found that they contained fewer ectoparasites than control nests, although breeding success did not differ between treatments (Shutler and Campbell 2007). This further suggests that cup lining material has only limited effects on parasite abundance.

In summary, it remains unclear whether green plant materials and feathers serve to reduce nest parasites. This uncertainty is further confounded by studies of other species that report contrasting findings. For example, the amount of green plant material in bonelli's eagle (*Hieraaetus fasciatus*) nests was negatively correlated with ectoparasite abundance (Ontiveros et al. 2008), whereas the number of nest lining feathers within barn swallow nests was negatively related to eggshell bacterial load (Peralta-Sanchez et al. 2010), thereby supporting an antiparasite function. Further, a fascinating study examined the function of cigarette butts incorporated into urban house sparrow and house finch (*Carpodacus mexicanus*) nests and found that the amount of cellulose acetate from the butts, which repels parasites, was negatively associated with the number of nest-dwelling parasites (Suárez-Rodríguez et al. 2013). However, green plant material in wood stork (*Mycteriaa mericana*) nests was found to provide insulation for chicks rather than to repel ectoparasites (Rodgers et al. 1988). Consequently, the exact function of green plant material and feathers remains unclear, and further studies are required to examine their function. They are probably multifunctional materials that limit parasite abundance, provide insulation, and play a role in sexual selection, and further studies that simultaneously examine these possibilities are required.

## Environmental Adjustment

The primary function of nests is to provide a suitable location for parents to lay their eggs and/or raise their offspring. The design of completed nests is known to influence the microclimate within the nest cup, thereby affecting the conditions experienced by both parents and offspring (Skowron and Kern 1980; Webb 1987; Ar and Sidis 2002; Dawson et al. 2011; Lambrechts et al. 2012; Ardia 2013). Nest microclimates that are suboptimal have negative impacts upon the growth and development of offspring (Lombardo et al. 1995). While parental behaviors, such as increased bouts of brooding, may help to regulate conditions within the nest so that they are within acceptable limits, such behaviors are likely to be energetically costly for parents (Reid et al. 2000; Deeming 2011). One way in which parents can mitigate this energetic demand is to alter the design of their nests to adjust to environmental conditions (Collias and Collias 1984; Webb 1987; Hansell 2000; Shimmin et al. 2002; Deeming 2011). Nevertheless, the construction of thermally optimal nests must be traded off against the associated energetic costs of nest construction (Skowron and Kern 1980; Mainwaring and Hartley 2013), and so variation in nest site selection and nest construction materials may result if parents adjust their nests to suit prevailing conditions (Webb 1987; Ar and Sidis 2002; Shimmin et al. 2002; McGowan et al. 2004). Consequently, the design of nests should vary adaptively in relation to predictable changes in environmental conditions with increasing spring temperatures, altitude, and latitude.

### Nest site selection

Prior to constructing a nest, one or both of the parents must decide on the location in which to construct the nest (Collias and Collias 1984). The selection of a suitable nest site is determined by a combination of five main factors: the availability of food for both parents and offspring, the risk of predation, the presence and behavior of conspecifics, the availability of suitable nest material, and the presence of a suitable ambient climate for raising offspring (Collias and Collias 1984; Hansell 2005). Ambient temperatures are usually lower than the optimal temperatures for offspring development, and empirical studies show that nests are located in sites that lose less heat than sites selected at random. Grasshopper sparrows (*Ammodramus savannarum*) and eastern meadowlarks (*Sturnella magna*) breeding in grasslands built domed nests that were orientated away from prevailing winds, and the orientation of the nests shifted temporally over the course of the breeding season as the direction of the prevailing winds changed (Long et al. 2009). Further, orange-tufted sunbirds (*Nectarinia osea*), horned larks (*Eremophila alpestris*), lark buntings (*Calamospiza melanocorys*), and McCown's longspurs (*Calcarius mccownii*) all selected nest sites that faced away from the prevailing winds, as well as being located away from direct sunshine during the middle part of the day, which prevented the nests from overheating (With and Webb 1993; Sidis et al. 1994; Hartman and Oring 2003). Lesser black-backed gulls (*Larus fuscus*) which nested adjacent to tall vegetation and were therefore sheltered from cold winds raised chicks that grew faster than chicks raised in more exposed nests which experienced cooler temperatures (Kim and Monaghan 2005). Further, an observational study of eider ducks (*Somateria mollissima*) showed that females breeding in sheltered nests experienced milder temperatures and laid larger clutches with higher hatching rates than females nesting in exposed nests at cooler temperatures. Then, when shelters were experimentally added to nests at exposed sites, experimental females had lower rates of mass loss than control females, although hatching success did not differ between the two treatments (D'Alba et al. 2009). By contrast, in arid environments, animals select sites that are cooler than randomly selected sites. Desert lark (*Ammomanes deserti deserti*) nests in an arid environment were found to be located adjacent to a bush or stone and to face north which provided shade from the midday sun (Orr 1970). Consequently, there is a large amount of empirical evidence to show that ground-nesting animals select sites that minimize heat loss in cool environments and prevent overheating in warm environments, thereby creating an optimal microclimate in which to raise offspring. However, those sites that create the optimal microclimate for offspring development may also be conspicuous to predators.

Two studies have examined the trade-off that parents face between selecting a site that creates a suitable microclimate for raising offspring and minimizing the risk of predation. Hoopoe larks (*Alaemon alaudipes*) breeding in a hot desert were found to select sites on gravel away from vegetation during the early part of the breeding season when temperatures were relatively cool. They then increasingly selected sites in shrubs as the season progressed and ambient temperatures increased, and while nest predation rates did not differ between the two sites, nests on the gravel experienced higher temperatures. This indicates that the exposed nest sites were preferred because the incubating adults could protect themselves from approaching predators during the early part of the breeding season, but were forced to move to more sheltered sites as the season progressed and temperatures increased (Tielman et al. 2008). Piping plovers (*Charadrius melodus*) preferentially laid their eggs on white pebbles that resembled the color of their eggs more closely than randomly available pebbles. Eggs that closely matched their adjacent pebbles suffered lower levels of predation, yet artificial nests constructed of randomly available pebbles warmed faster and were warmer than plover nest pebbles, with temperatures inside nests being about 2–6°C cooler than surrounding substrates. The nest sites rapidly lost heat when they were not incubated by an adult, which suggests that pebble selection is a trade-off between maximizing heat reflectance to improve egg microclimate and minimizing the conspicuous contrast of eggs and surrounding substrates (Mayner et al. 2009).

Spotted owls (*Strix occidentalis*) were found to select those nesting holes that provided the most suitable microclimate for incubating parents and developing offspring. This is important for the owls as they live in northern regions of America that are characterized by inclement weather during the nesting season, and the owl pairs which chose sites out of the wind had higher reproductive success than pairs in exposed sites (Rockweit et al. 2012). The orientation of Gila Woodpecker nests changed temporally throughout the year. In the breeding season, northerly facing holes reduced the levels of water loss from nests in the hot summer months, while warmer south-facing nests reduced energy expenditures during the cold winter months (Inouye et al. 1981). The thermal properties of spiny-cheeked honeyeater (*Acanthagenys rufogularis*) and yellow-throated miner (*Manorina flavigula*) nests were studied across three wind speeds. Nest dimensions differ between the species, despite the adults having similar body masses, although the nest conductance of both species nests is comparable. The study found that the rate of heat loss from nests increased in both species as wind speed increased and as a result of forced convection through the nest, incubating parents would be required to double their heat production to maintain a suitable microclimate within the nest (Heenan and Seymour 2012). Nestbox-breeding prothonotary warbler (*Protonotaria citrea*) pairs that nested early in the season, when ambient temperatures were low, preferentially selected those nestboxes which had the highest ambient temperatures. Pairs that nested late in the season when ambient temperatures were warm preferentially selected those nestboxes which had the lowest ambient temperatures (Blem and Blem 1994).

The importance of nest sites in creating optimal microclimates for offspring development was also supported in an interspecific study (Burton 2007). A comparative study of seven North American and European bird species found a trend toward nests being north-facing at lower latitudes and eastward- or southward-facing farther north. At southern latitudes, the requirement for shade results in birds selecting northward orientations, at mid-latitudes, predominantly easterly orientations reflect the balance between the benefits of warmth in the early morning and shade in the afternoon, while at northern latitudes, nests are orientated southwards to gain warmth throughout the day (Burton 2007). Therefore, while it is clear that animals create the optimal microclimate for offspring development by selecting sites that either conserve or lose heat more efficiently than sites selected at random, the microclimates within nests can be further enhanced by the addition of various construction materials (Hansell 2005).

### Nest construction materials

The majority of nests are differentiated structures that are constructed from a variety of materials which can generally be classified as being either structural materials or lining materials. While structural materials make up the general shape of the nest and provide structural support for the parents and offspring, lining materials generally create a suitable microclimate in which parents can raise their offspring (Hansell 2000, 2005). The exact function of structural materials is not yet fully understood because while an interspecific study of Australian birds that build cup-shaped nests suggested that structural support for the eggs and incubating parents was the primary factor driving nest design (Heenan and Seymour 2011), other studies have shown that structural materials provide thermoregulatory benefits. Illustratively, the nests of white-crowned sparrows (*Zonottrichia leucophrys*) block out 96–99% of air currents to which they are exposed (Kern 1984). Further, the enclosed nests of cactus wrens (*Campylorhynchus brunneicapillus*) moderate the nest environment under widely varying environmental conditions by both retaining heat during cold weather and by shading the nest contents from direct sunlight during hot weather (Ricklefs and Hainsworth 1969). Meanwhile, several studies have examined the function of the structural materials in the nests of sociable weavers (*Philetairus socius*), where nests are huge structures that contain the individual nesting chambers of colonies of birds that sometimes compromise a hundred or more pairs. The enormous nests have been found to ameliorate the impact of low temperatures (White et al. 1975) and significantly reduce the metabolic expenditure by colony members (Bartholomew et al. 1976). However, the thermal benefits of individual nests vary in relation to the size of the communal nest and the position of individual nest chambers within it. This has important consequences for the fitness of individuals as higher quality individuals occupy those nests that maintain heat with the most efficiency (Van Dijk et al. 2013). Therefore, while a comparative study indicates that structural materials provide structural support for the parents and offspring (Heenan and Seymour 2011), empirical studies also show that structural materials provide thermoregulatory benefits for the parents and offspring (Kern 1984; Bollazzi and Roces 2010). Consequently, further studies are required to elucidate the function of structural materials in nests. By contrast, there is little doubt that the function of nest lining materials is to provide thermoregulatory benefits to the parents and offspring within the nest cup.

Among ground-nesting birds, one interspecific study of six species of Arctic breeding shorebirds showed that smaller species, with greater surface-area-to-volume ratios, created nest scrapes with greater amounts of nest lining material than larger species, thereby demonstrating that smaller species invest more in the insulation of their nests than larger species (Tulp et al. 2012). Pectoral sandpipers (*Calidris melanotos*) have been shown to excavate a scrape and use lining material, which reduced the rate at which the nests lost heat by 9% and 25%, respectively. Hence, lined scrapes insulate clutches much more efficiently than unlined scrapes (Reid et al. 2002).

Many birds line their nests with feathers (Calvelo et al. 2006; Liljeström et al. 2009), which is advantageous as when the insulation properties of a range of commonly used lining materials were tested in the laboratory, feathers were found to provide the most insulation to nests, while grasses provided the least (Hilton et al. 2004). The most comprehensive studies of the function of feathers as a nest lining material come from studies of tree swallows. Meanwhile, nestlings in experimental nests with added feathers were structurally larger at prefledging than nestlings in control nests, suggesting that feathers provided thermal benefits that resulted in increased nestling growth (Dawson et al. 2011). This conclusion was supported in an observational study that demonstrated that nests with more feathers and with deeper nest cups cooled at slower rates than nests with fewer feathers and shallow nest cups (Windsor et al. 2013). Experimental nests, in which feathers were removed, contained nestlings that were lighter and had shorter tarsi and wing chords than nestlings in control nests. Ectoparasite abundance was unaffected by the removal of feathers, but experimental nests had higher fledging success which indicates that the insulation quality of feathers increases reproductive success (Lombardo et al. 1995). In another study, nestlings in experimental nests where feathers were removed had lower rates of mass, tarsus, and wing length growth and higher infestations of mites and lice, when compared to nestlings in control nests. Therefore, feathers benefitted nestlings directly by keeping them warm and indirectly by facilitating higher growth rates, but once again, there was no reduction in the nestling's exposure to detrimental ectoparasites (Winkler 1993).

The fitness consequences of varying temperatures within nests have also been examined in tree swallows. In experimentally cooled nests, incubating females reduced the intensity of their incubation behaviors, which resulted in extended incubation times and lighter nestlings with weaker immune systems, when compared to nestlings raised in control nests (Ardia et al. 2008). Meanwhile, experimentally heated nests resulted in females maintaining a higher body condition than control females, which resulted in them provisioning their nestlings at higher rates and raising heavier nestlings than females in control broods (Peréz et al. 2008). In multibrooded starlings, those pairs that had their nests heated during the incubation phase of their first brood had higher levels of fledging success in that first brood and higher levels of hatching success in second broods, when compared to control pairs (Reid et al. 2000). Consequently, there is good evidence to suggest that nest lining materials, such as feathers, serve to create suitable microclimates in which to raise offspring and not to provide protection from ectoparasites. However, environmental conditions are not stable over temporal or spatial timescales, and the design of nests must vary accordingly.

### Spring temperatures

At temperate latitudes, nest design should vary in relation to increasing ambient temperatures as spring advances. A series of observational studies have examined seasonal variation in blue tit and great tit nest characteristics (Mainwaring and Hartley 2008; Britt and Deeming 2011; Deeming et al. 2012). The nest-building period of blue tits was found to decrease seasonally, probably because later pairs needed to build their nests rapidly in order to synchronize the time of maximal nestling food demand with the period of maximal availability of their winter oak moth (*Operophtera brumata*) caterpillar food supply. Despite this pattern, there was no seasonal trend in the mass of nests, but there were seasonal changes in nest composition. The mass of the nests' moss base showed no seasonal variation, but there was a seasonal decline in the mass of the cup lining material (Mainwaring and Hartley 2008). A similar study reported that the amount of both animal- and plant-derived materials decreased with increasing spring temperatures as spring progressed in blue tits, but not in great tits (Britt and Deeming 2011). A further study showed that the mass of nest cup lining materials decreased as spring temperatures increased along a latitudinal gradient in both blue tits and great tits (Deeming et al. 2012). Together, these studies suggest that female blue tits and great tits are able to gauge environmental conditions and selectively adjust the cup lining component of their nests to reflect increasing ambient temperatures as spring progresses.

In Chilean swallows (*Tachycineta meyeni*), there was a negative association between the number of feathers added to nests and the average daily ambient temperatures, which increased as spring progressed (Liljeström et al. 2009). The hatching success of the eggs was not associated the number of feathers at the start of laying or at the end of incubation, and there was no association between the number of feathers and the average weight of the nestlings at the prefledging stage. Consequently, the swallows make temporal adjustments to the number of feathers that they add to nests over the course of the breeding season (Liljeström et al. 2009).

An observational study of long-tailed tits (*Aegithalos caudatus*) showed that the mass of feathers used as cup lining materials declined through the breeding season, but there was no seasonal decrease in nest insulation quality because of increasing ambient temperatures. Then, in an experimental study where feathers were added to experimental nests at an early stage of the lining phase of nest construction, the total mass of feathers in experimental nests was comparable to that in control nests, and there was no significant difference in the insulation quality of nests. The experimental provisioning of feathers at experimental nests meant that parents at experimental nests collected approximately 50% fewer feathers. This reduction in effort is insightful as there was no significant effect on the duration over which feathers were collected, suggesting that the seasonal decline in feather mass was due to long-tailed tits adjusting feather mass to environmental conditions. This also suggests that feathers were not a limiting resource (McGowan et al. 2004), which is consistent with a previous study that found that when a range of woodland passerine birds, including long-tailed tits and blue tits, were supplied with feathers, they were barely used as nest material (Hansell and Ruxton 2002). Therefore, these studies provide good evidence that nest design varies adaptively in relation to predictable temporal increases in ambient temperatures as spring advances, and an experimental study (McGowan et al. 2004) suggests that these patterns are not a function of the availability of feathers or time constraints).

### Altitude

One study has examined nest site selection and nest design in relation to decreasing ambient temperatures as altitude increases. On Hawaii, the nests of Common Amakihi (*Hemignathus virens virens*), which are small finches in the Hawaiian honeycreeper subfamily (Whittow and Berger 1977), were compared at two sites at different altitudes (Kern and van Riper 1984). Common Amakihi's breed during the wet season and so irrespective of altitude, all nests were located within tree canopies so that they were protected from the rain. However, nests at higher altitudes were more likely to be placed higher in the canopies and closer to the edge of trees than nests at lower altitudes, so that they would be warmed by radiant solar energy. Common Amakihi's breeding at higher altitudes also built nests with denser, but not thicker, walls that also contained more cup lining material. This resulted in them having higher insulation capacity, but being less porous and slower drying, than nests built by conspecifics at lower altitudes (Kern and van Riper 1984). This study provides good evidence that birds vary the design and structure of their nests in relation to decreasing ambient temperatures as altitude increases, but further studies are required to assess the generality of this trend.

Therefore, given that nest design varies adaptively in relation to predictable changes in temperature at small spatial scales, such as those found within a study area, then nest design should also be expected to vary adaptively over large spatial scales, such as with decreasing ambient temperatures as latitude increases.

### Latitude

An interspecific study of passerine birds in Europe demonstrated that those species which breed relatively early and hence, at lower ambient temperatures, were more likely to add feathers as nest lining material to their nests than later breeding species (Møller 1984). Meanwhile, an intraspecific study showed that citrine wagtails (*Motacilla citreola*) breeding at the northerly part of their breeding range lined their nests with feathers while their more southerly breeding conspecifics did not (Møller 1984). Other cup nesting birds also show latitudinal variation in nest composition. An examination of the nest structures of yellow warblers (*Dendroica petechia*) breeding in northern and southern Canada showed that birds breeding further north built larger, less porous nests that retained heat better but also absorbed more water and then took longer to dry than nests from the south (Briskie 1995; Rohwer and Law 2010). Also, American robin (*Turdus migratorius*), yellow warbler, and *Carduelis* finches nests were heavier and had thicker nest walls in northern Canada than in southern Canada (Crossman et al. 2011). Patterns of nest site selection of northern oriole (*Icterus spp*) nests also varied with latitude. Nests in the north were built on thinner branches, presumably to make them less accessible to squirrels. Nests in the south are better protected from the sun as they have a smaller opening and more spacious than those further north (Schaeffer 1976). The nest insulatory properties of northern oriole nests also varied with latitude, being better insulated in the north than in the south (Schaeffer 1980). Elsewhere, common blackbirds living in cooler environments at higher latitudes within Great Britain built nests with thicker walls and consequently, greater insulatory properties, than conspecifics living in warmer environments at lower latitudes (Mainwaring et al. 2014).

Among hole nesting birds, the mass of the cup lining material and nest insulatory properties of blue tit and great tit nests decreased with increasing spring temperatures as latitude decreased in Great Britain (Mainwaring et al. 2012). As spring temperatures increased with decreasing latitude, the mass of the nest base material did not vary in either species, while the mass of the cup lining material and nest insulatory properties decreased in both species. This suggests that in response to increasing temperatures, the breeding female reduces the mass of the cup lining material, thereby maintaining an appropriate microclimate for incubating and brooding (Mainwaring et al. 2012).

In summary, these results indicate that the decrease in the mass of the nest cup lining material in birds' nests may be counteracting increasing spring temperatures to create an appropriate microclimate for both parents and offspring. There are now several studies that report the fine-scale adjustment of nest cup lining material in response to ambient temperatures in birds (e.g., McGowan et al. 2004; Mainwaring and Hartley 2008; Britt and Deeming 2011), which is important as a recent study has shown that the nest microclimate has important consequences for the body condition of both parents and chicks in tree swallows (Peréz et al. 2008). Further research is required to investigate how nest construction reflects the thermoregulatory needs of the incubating adult. Moreover, nest lining material has been shown to have sexual (Sanz and García-Navas 2011) and other nonthermoregulatory (Mennerat et al. 2009b) functions, and further research could usefully examine the functionality of nest lining. To summarize, there is a reasonable amount of evidence to show that both hole nesting and open-cup nesting species systematically vary the design of their nests in response to large-scale latitudinal variation in ambient temperatures.

## Conclusions and Further Work

Our understanding of the design and function of birds' nests has increased considerably in recent years and the evidence suggests that nests have several nonmutually exclusive functions. Therefore, we conclude that far from being simple receptacles for eggs and/or offspring, the design and function of birds' nests is far more sophisticated than previously realized. Nevertheless, there are still several areas that are likely to be fruitful for future research. First, both natural and sexual selection appear to influence nest design, yet while natural selection selects for smaller nests, sexual selection selects for larger nests. One recent study (Sergio et al. 2011) strongly suggests that natural and sexual selection are directly traded off against each other and further studies should examine the resolution of these conflicting requirements. Second, empirical studies examining the design and function of birds' nests are distributed nonrandomly with respect to their ecology, with the vast majority of studies involve small hole nesting passerines which breed inside nestboxes (Lambrechts et al. 2010). This bias is understandable as species such as blue tits and starlings are logistically easy to study, yet future studies could usefully assess the generality of these findings by studying open-cup nesting species. This is important as open-cup nesting birds are likely to be under very different selection pressures to hole nesting birds. Third, there is concern that climate change may negatively affect nest-building animals. We therefore urge future studies to examine how climate change may affect nest-building behaviors and the design of the completed nest.
